# Impact of prior Dengue immunity on Zika vaccine protection in rhesus macaques and mice

**DOI:** 10.1371/journal.ppat.1009673

**Published:** 2021-06-25

**Authors:** Rafael A. Larocca, Peter Abbink, John D. Ventura, Abishek Chandrashekar, Noe Mercado, Zhenfeng Li, Erica Borducchi, Rafael A. De La Barrera, Kenneth H. Eckels, Kayvon Modjarrad, Michael P. Busch, Nelson L. Michael, Dan H. Barouch

**Affiliations:** 1 Center for Virology and Vaccine Research, Beth Israel Deaconess Medical Center, Harvard Medical School, Boston, Massachusetts, United States of America; 2 Walter Reed Army Institute of Research, Silver Spring, Maryland, United States of America; 3 Vitalant Research Institute, San Francisco, California United States of America; 4 Department of Laboratory Medicine, University of California San Francisco, San Francisco, California, United States of America; 5 Ragon Institute of Massachusetts General Hospital, Massachusetts Institute of Technology, and Harvard, Cambridge, Massachusetts, United States of America; Duke-National University of Singapore, SINGAPORE

## Abstract

Pre-existing immunity to flaviviruses can influence the outcome of subsequent flavivirus infections. Therefore, it is critical to determine whether baseline DENV immunity may influence subsequent ZIKV infection and the protective efficacy of ZIKV vaccines. In this study, we investigated the impact of pre-existing DENV immunity induced by vaccination on ZIKV infection and the protective efficacy of an inactivated ZIKV vaccine. Rhesus macaques and mice inoculated with a live attenuated DENV vaccine developed neutralizing antibodies (NAbs) to multiple DENV serotypes but no cross-reactive NAbs responses to ZIKV. Animals with baseline DENV NAbs did not exhibit enhanced ZIKV infection and showed no overall reduction in ZIKV vaccine protection. Moreover, passive transfer of purified DENV-specific IgG from convalescent human donors did not augment ZIKV infection in *STAT2* ^-/-^ and BALB/c mice. In summary, these results suggest that baseline DENV immunity induced by vaccination does not significantly enhance ZIKV infection or impair the protective efficacy of candidate ZIKV vaccines in these models. These data can help inform immunization strategies in regions of the world with multiple circulating pathogenic flaviviruses.

## Author summary

Whether the induction of anti-Dengue immunity by vaccination affects the protective efficacy of Zika virus (ZIKV) vaccines is an important consideration for public health programs aimed at controlling Dengue and Zika transmission. Here, we report the impact of previous anti-Dengue virus (DENV) immunity elicited by both live-attenuated tetravalent (TDENV-LAV) or single-serotype inactivated DENV vaccination on subsequent ZIKV vaccine efficacy in both rhesus macaques and mice. In macaques and mice, prior anti-DENV vaccination did not generate cross-reactive neutralizing antibodies against ZIKV. Previous immunization with TDENV-LAV showed no significant enhancement of ZIKV infection or reduced the protective efficacy of a subsequent immunization with candidate ZIKV vaccines. In addition, ZIKV viral loads were not enhanced following ZIKV challenge of *STAT2* ^*-/-*^ mice previously passively transferred with anti-DENV IgG. These results suggest that prior immunization with DENV vaccines have a minimal impact on ZIKV disease enhancement and do not impact the overall protective efficacy of subsequent ZIKV immunization in both macaques and mice.

## Introduction

Dengue virus serotypes 1–4 (DENV1-4) and Zika virus (ZIKV) are two closely related pathogenic flaviviruses transmitted by mosquitos of the genus *Aedes*. DENV infection can lead to devastating clinical outcomes, and although most cases are asymptomatic, severe cases of dengue fever can lead to vascular leak syndrome and shock [1]. ZIKV infection predominantly leads to self-limiting disease that includes mild rashes, arthralgia, myalgia, and fever [2]. However, the World Health Organization declared the ZIKV pandemic a public health emergency of international concern in 2016 following an increased number of microcephaly and Guillain Barre syndrome cases, particularly in northeastern Brazil [3–6].

DENV and ZIKV co-circulate in human populations in tropical and subtropical regions, and efforts to develop safe and effective vaccines against both viruses are currently underway [7]. Whether immunity against one of these flaviviruses protects against or exacerbates infection with the other flaviviruses remains unclear. It is well described that previous flavivirus exposure can influence the severity of subsequent flavivirus infections. Primary infection with one DENV serotype can generate life-long immunity against itself, whereas secondary exposure with a heterotypic serotype can lead to severe dengue disease [8,9]. Evidence suggests that this phenomenon is mainly caused by cross-reactive poorly neutralizing anti-DENV antibodies that engage Fcγ receptors on target myeloid cells, facilitating viral uptake and enhancing cellular infection, a process known as antibody-dependent enhancement (ADE) [9–12]. Antibodies raised against DENV can cross-react to ZIKV to varying degrees, raising concern that previous exposure to DENV or other flaviviruses may enhance ZIKV infection [13–16]. Several recent studies have reported that ZIKV infection was enhanced in the presence of convalescent plasma derived from DENV-infected patients *in vitro*, and passive transfer of anti-DENV immune sera exacerbated ZIKV infection in *STAT2*^*-/-*^ knockout mice [13,17].

Whether co-infection enhances disease in natural settings is still unclear, and assessment of secondary flavivirus infections in large human cohorts is currently ongoing. A large epidemiological study of urbanites from Salvador, Brazil, reported a reduced risk of ZIKV infection and symptom manifestation in people with high pre-existing anti-DENV titers [18]. Additionally, a study following a pediatric cohort in Nicaragua suggested that high titers of pre-existing anti-DENV protected against secondary exposure to DENV-1 and DENV-3 serotypes as well as ZIKV. However, moderate titers induced by previous ZIKV or DENV infection increased DENV-2 and DENV-3 infection [19]. High pre-existing anti-DENV titers were associated with reduced risk, whereas intermediate anti-DENV or anti-ZIKV titers were associated with increased risk of DENV-2 and DENV-3 disease [20].

These findings show that prior flavivirus immunity may modify secondary flavivirus infections, complicating vaccination efforts against both DENV and ZIKV. Moreover, it is unclear how pre-existing anti-DENV immunity impacts subsequent ZIKV infection, as it is also unclear whether it impairs ZIKV vaccine efficacy. In this report, we induced anti-DENV immunity in both rhesus macaques and mice through vaccination and investigated whether pre-existing anti-DENV immunity influences ZIKV infection or ZIKV vaccine protection. We observed that primary anti-DENV immunity did not impact ZIKV infection or ZIKV vaccine efficacy in vivo. Moreover, passive transfer of anti-DENV IgG did not enhance ZIKV infection in either *STAT2—*^/-^ knockout or wild-type BALB/c mice. These findings show that baseline anti-DENV NAbs did not significantly enhance ZIKV infection or attenuate ZIKV vaccine efficacy in these models.

## Results

### Cross-reactive DENV and ZIKV NAb responses following vaccination in rhesus macaques

**Fig 1:**
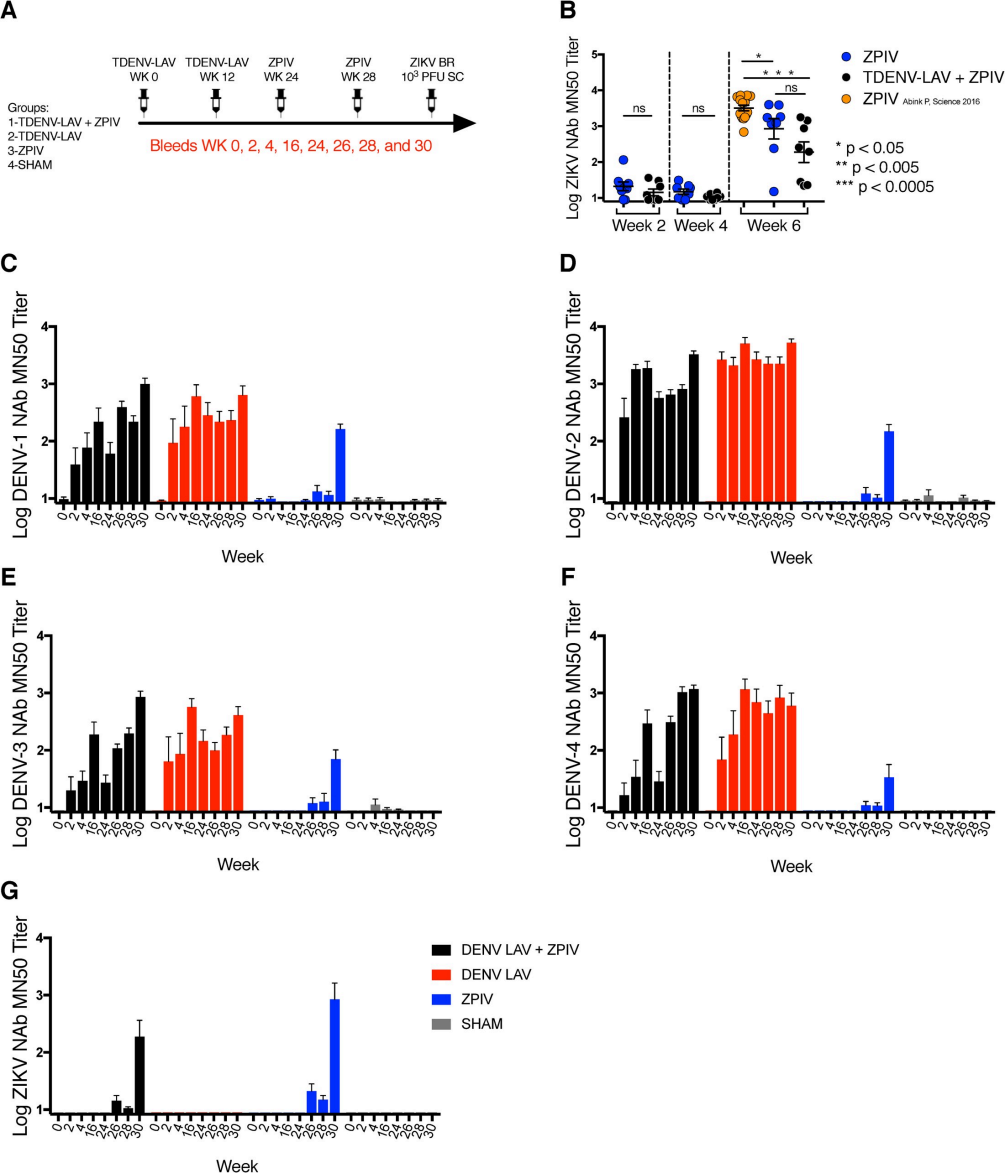
Induction of Neutralizing antibodies by TDENV-LAV and ZPIV vaccines in rhesus monkeys. (A) Study Schematic Design. Monkeys (distributed into 4 groups, n = 8 per group) were immunized intramuscularly (i.m.) on weeks 0 and 12 with 10^3^ PFU of the live attenuated tetravalent DENV vaccine (TDENV-LAV) and vaccinated on weeks 24 and 28 with 5 μg of the purified inactivated ZIKV (ZPIV) and challenged s.c. with 10^3^ PFU ZIKV-BR 4 weeks after vaccination. (B) Log ZIKV-specific MN50 titers in GMP ZPIV (blue dots), TDENV-LAV+ZPIV (black dots), and the research grade ZPIV vaccinated rhesus monkeys at week 6 post ZPIV prime (30 weeks bleed time point, orange dots). DENV microneutralization assay for DENV-1 (C), DENV-2 (D), DENV-3 (E), DENV-4 (F), and ZIKV (G). Data is representative of one experiment with 8 animals per group. Each dot represents an individual monkey. *P* values were calculated using a *Mann-Whitney U test*. Mean ± SEM is shown.

First, we interrogated the cross-reactivity profile of vaccine-induced NAbs responses induced by a tetravalent DENV vaccine (TDENV-LAV) and the clinical GMP lot of a purified inactivated Zika virus vaccine (ZPIV). A cohort of 32 rhesus macaques was randomly distributed in 4 experimental groups (n = 8 per group): (1) in the TDENV-LAV + ZPIV Group, animals were pre-immunized with 10^3^ plaque-forming unit (PFU) TDENV-LAV and then received two doses of 5 μg ZPIV, (2) in the TDENV-LAV Group, animals were pre-immunized with TDENV-LAV vaccine only, (3) in the ZPIV Group, animals were immunized with ZPIV vaccine only, and (4) in the Sham Group, animals received saline (Fig 1A). TDENV-LAV and ZPIV vaccinations were administered at 12- and 4-week intervals, respectively. Prior to vaccination, animals were screened for previous flavivirus exposure. NAbs to DENV serotypes (1, 2, 3, and 4), West Nile virus (WNV), and yellow fever virus (YFV) were detected in a subset of animals, although none of the animals had detectable NAbs for ZIKV (Table 1). We also compared the immunogenicity of the GMP batch of ZPIV with a previously published research-grade batch of ZPIV [21,22]. ZIKV-specific NAb titers were slightly lower with the GMP batch of ZPIV compared with research-grade ZPIV at 6 weeks post ZPIV prime (week 30 time point in Fig 1A and 1B). We then evaluated the DENV NAbs responses induced by TDENV-LAV immunization within each vaccination group. We observed robust NAbs responses against all four DENV serotypes within two weeks post-vaccination (Fig 1C–1F). Notably, previous TDENV-LAV vaccination alone did not induce cross-reactive NAb against ZIKV, and anti-ZIKV NAbs titers were detected only after ZPIV immunization (Fig 1G). In contrast, ZPIV vaccination led to cross-reactive NAbs titers against DENV-1, 2, 3, and 4 after the ZPIV boost (Fig 1C–1F).

**Table 1:** Log titer of previous flavivirus seropositivity in animals used in this study.

VACCINE	Monkey ID	Gender	DENV-1	DENV-2	DENV-3	DENV-4	JEV	YFV	WNV	ZIKV
TDENV LAV + ZPIV	T737	Female	<1	<1	<1	<1	<1	<1	1.89	<1
TDENV LAV + ZPIV	T738	Female	<1	<1	<1	<1	<1	<1	<1	<1
TDENV LAV + ZPIV	T739	Female	<1	<1	<1	<1	<1	<1	<1	<1
TDENV LAV + ZPIV	T740	Female	<1	<1	<1	<1	<1	<1	<1	<1
TDENV LAV + ZPIV	T741	Female	<1	<1	<1	<1	<1	<1	<1	<1
TDENV LAV + ZPIV	T742	Female	<1	<1	<1	<1	<1	<1	<1	<1
TDENV LAV + ZPIV	T743	Female	1.26	<1	<1	<1	<1	<1	<1	<1
TDENV LAV + ZPIV	T744	Female	<1	<1	<1	<1	<1	<1	<1	<1
ZPIV	T745	Female	<1	<1	<1	<1	<1	<1	1.99	<1
ZPIV	T746	Female	<1	<1	<1	<1	<1	<1	<1	<1
ZPIV	T747	Female	<1	<1	<1	<1	<1	<1	<1	<1
ZPIV	T748	Female	<1	<1	<1	<1	<1	<1	<1	<1
ZPIV	T749	Female	<1	<1	<1	<1	<1	<1	<1	<1
ZPIV	T750	Female	<1	<1	<1	<1	<1	<1	<1	<1
ZPIV	T751	Female	1.15	<1	<1	<1	<1	<1	2.97	<1
ZPIV	T752	Female	0.95	<1	<1	<1	<1	<1	0.95	<1
TDENV LAV	T753	Female	1.04	<1	<1	<1	1.11	<1	2.79	<1
TDENV LAV	T754	Female	<1	<1	<1	<1	<1	<1	0.95	<1
TDENV LAV	T755	Female	<1	<1	<1	<1	<1	<1	2.02	<1
TDENV LAV	T756	Female	<1	<1	<1	<1	<1	<1	<1	<1
TDENV LAV	T757	Female	<1	<1	<1	<1	<1	<1	<1	<1
TDENV LAV	T758	Female	<1	<1	<1	<1	<1	<1	<1	<1
TDENV LAV	T759	Female	<1	<1	<1	<1	<1	<1	<1	<1
TDENV LAV	T760	Female	<1	<1	<1	<1	<1	<1	<1	<1
SHAM	T761	Female	<1	<1	<1	<1	<1	<1	<1	<1
SHAM	T762	Female	<1	1.04	<1	<1	<1	<1	<1	<1
SHAM	T763	Female	<1	<1	<1	<1	<1	<1	<1	<1
SHAM	T764	Female	1.18	<1	<1	<1	1.23	<1	2.41	<1
SHAM	T776	Female	<1	<1	<1	<1	<1	<1	<1	<1
SHAM	T777	Male	<1	<1	<1	<1	<1	<1	<1	<1
SHAM	T778	Female	<1	<1	<1	<1	<1	<1	<1	<1
SHAM	T779	Female	<1	<1	<1	<1	<1	<1	<1	<1

### Baseline DENV-specific immunity does not lead to significant enhancement of ZIKV infection or abrogate ZIKV vaccine efficacy in rhesus macaques

**Fig 2:**
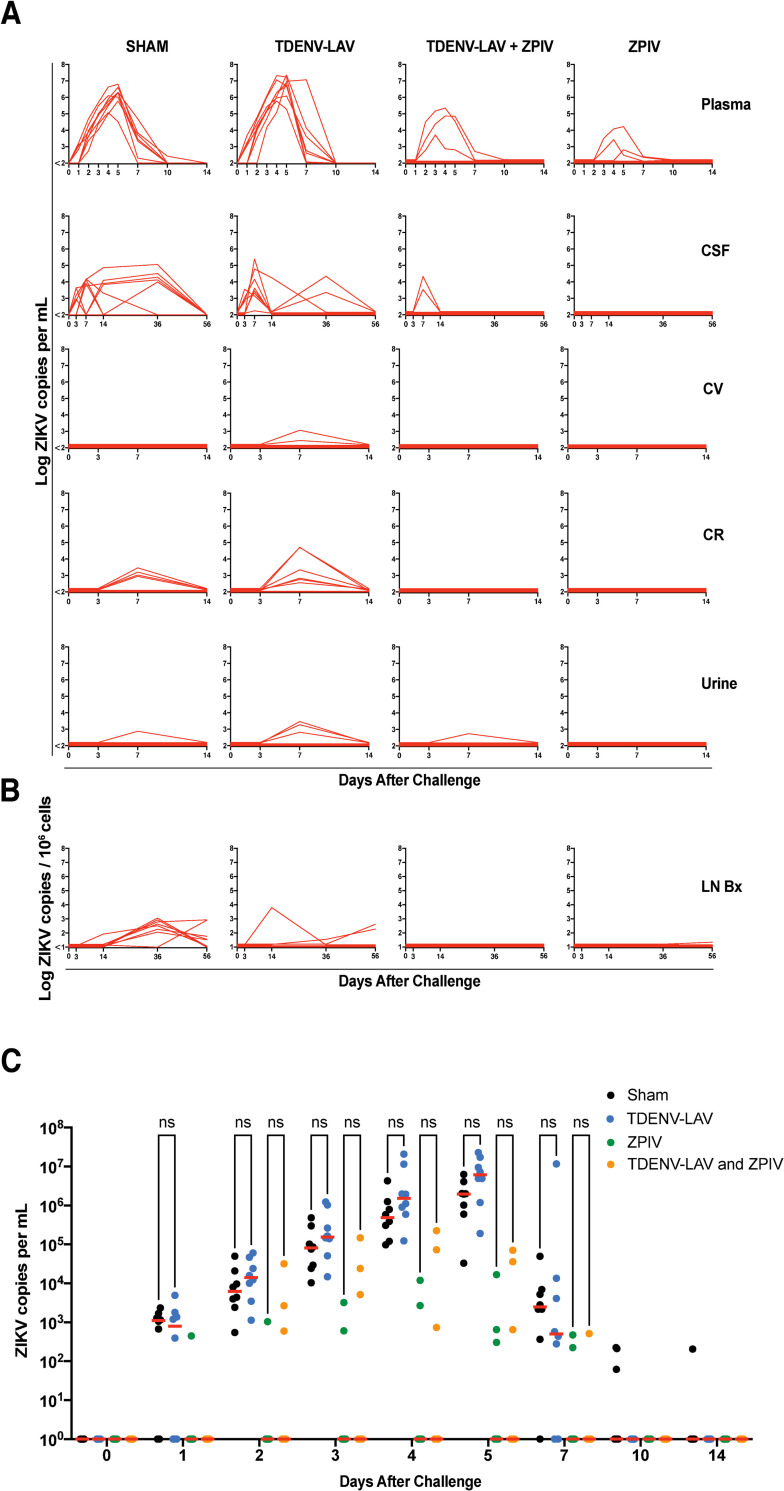
Previous DENV-Immunity induced by vaccination does not enhance ZIKV disease in rhesus monkeys. Monkeys (n = 32) were immunized intramuscularly (i.m.) on weeks 0 and 12 with 10^3^ PFU of the live attenuated tetravalent DENV vaccine (TDENV-LAV) and vaccinated on weeks 24 and 28 with 5 μg of the purified inactivated ZIKV (ZPIV) and challenged s.c. with 10^3^ PFU ZIKV-BR 4 weeks after vaccination. (A) ZIKV mRNA viral loads in plasma, cerebrospinal fluid (CSF), cervicovaginal swab (CV), colorectal swab (CR), and urine. (B) ZIKV mRNA viral loads in lymph-node. Data is representative of one experiment with 8 animals per group. Each line represents an individual monkey. (C) ZIKV plasma viremia in monkeys from all vaccine groups after ZIKV challenge. Each animal is represented as a dot and significance was calculated using a two-way analysis of variance using Tukey’s test for multiple comparisons.

We next assessed whether anti-DENV immunity induced by pre-immunization with TDENV-LAV would influence ZPIV vaccine-mediated protective efficacy against ZIKV challenge. At week 30, animals in all four vaccine groups were challenged with 10^3^ PFU of ZIKV-BR by the subcutaneous (s.c.) route [21,22]. ZIKV viral loads were measured by RT-PCR in plasma, cerebrospinal fluid (CSF), cervicovaginal swabs (CV), colorectal swabs (CR), urine, and lymph node biopsies (LN Bx) (Fig 2A and 2B). Macaques pre-immunized with TDENV-LAV exhibited no significant difference in ZIKV viral loads in multiple anatomical sites compared with those that did not have baseline DENV immunity (Fig 2). Vaccine protection was observed in both groups that received ZPIV compared with groups that were not immunized with ZPIV, regardless of TDENV-LAV pre-immunization. Partial protection was seen in 3 of 8 animals due to the presence of breakthrough virus in each ZPIV group (Fig 2A and 2B). When considering all cases of breakthrough viremia, differences in ZIKV viremia between the Sham and TDENV-LAV groups as well as the TDENV-LAV + ZPIV and the ZPIV alone groups were not significant (Fig 2C). We speculate that the lower degree of protective efficacy observed with ZPIV in this study as compared with prior studies may reflect the reduced potency of the GMP batch of ZPIV compared with prior research-grade batches of ZPIV (Fig 1B) [21]. Taken together, these data suggest that baseline DENV immunity induced by TDENV-LAV does not significantly enhance subsequent ZIKV infection and does not affect ZIKV vaccine protective efficacy.

### Baseline DENV-specific immunity does not lead to enhanced ZIKV infection or abrogate ZIKV vaccine efficacy in mice

We next assessed the level of cross-reactive anti-ZIKV endpoint titers generated by prior DENV vaccination. We primed BALB/c mice with 1 μg of one of the individual serotypes DENV-1, DENV-2, DENV-3, and DENV-4 purified inactivated virus (PIV) vaccines or 10^2^ PFU of the fully formulated TDENV-LAV vaccine and boosted with one of the following ZIKV vaccines (a) 10^9^ particles of RhAd52-M.ENV, (b) 1 μg GMP ZPIV, (c) 50 μg of DNA-M-ENV, or (d) no ZIKV vaccine control (sham). Mice were inoculated i.v. in these studies since we observed a statistically significant increase in peak viremia following the i.v. route as opposed to the s.c. route (S1 Fig). Mice vaccinated with the DENV-1 PIV vaccine exhibited relatively low levels of cross-reactive anti-ZIKV endpoint titers, and DENV-2 PIV vaccinated mice showed higher cross-reactive anti-ZIKV endpoint titers than was measured in mice preimmunized with DENV-1 PIV (S2 Fig). Anti-ZIKV cross-reactive endpoint titers were highest in mice preimmunized with DENV-3 and DENV-4 PIV, and mice immunized with TDENV-LAV displayed almost undetectable anti-ZIKV endpoint titer (S2 Fig).

**Fig 3:**
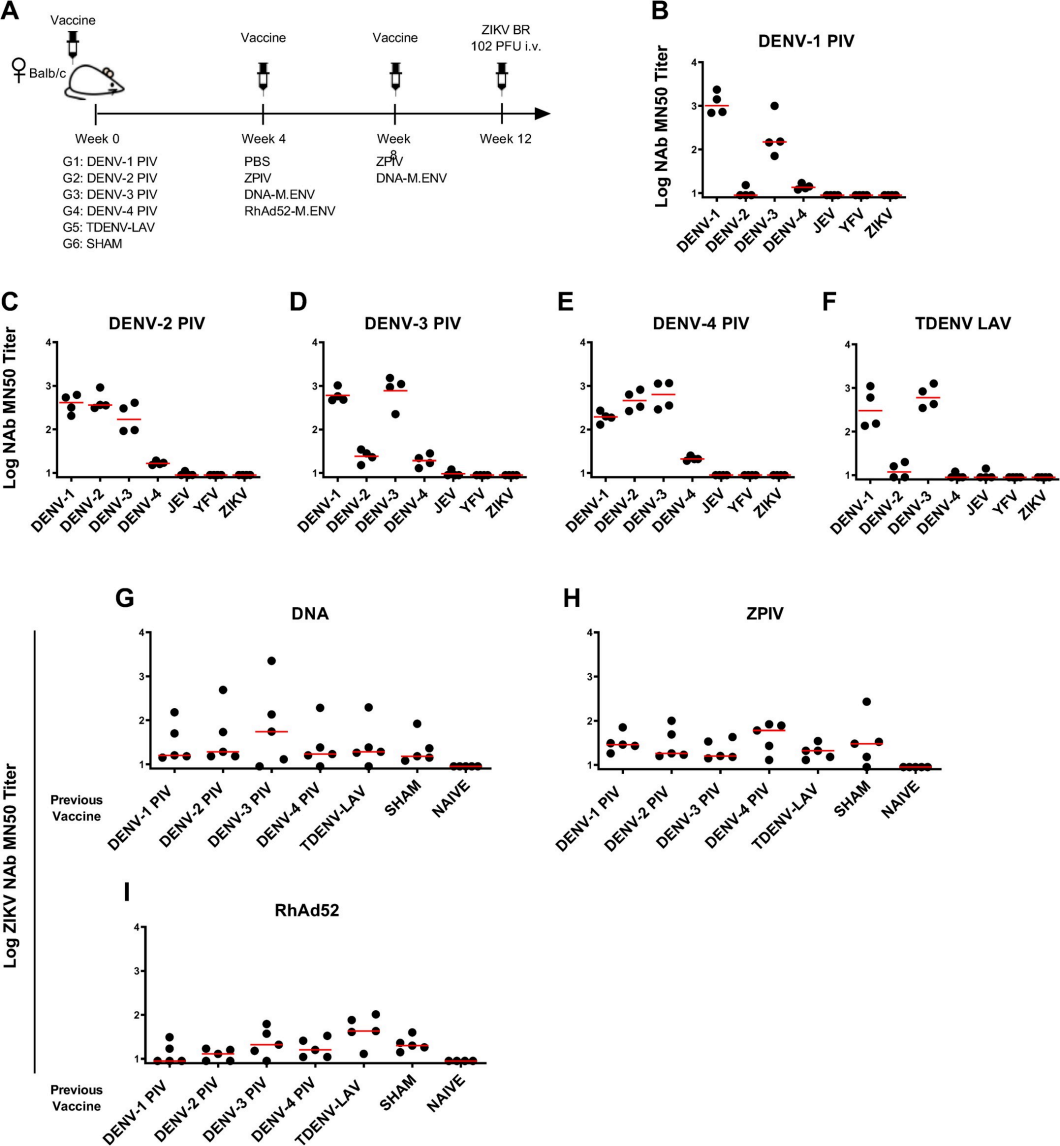
Induction of Neutralizing antibodies by TDENV-LAV, DENV-PIV and ZPIV vaccines. (A) Study Schematic Design. Mice (n = 5 mice per group) were immunized intramuscularly (i.m.) on weeks 0 with 1 μg of each DENV serotype of the purified inactivated vaccine (DENV-PIV) or 10^2^ PFU of the live attenuated tetravalent DENV vaccine (TDENV-LAV) and vaccinated on weeks 4 and 8 with 50 μg of the DNA-M.ENV vaccine and 1 μg of the purified inactivated ZIKV (ZPIV). Mice received a single immunization with 10^9^ vp of the RhAd52-M.ENV vaccine. Mice were challenged i.v. with 10^2^ PFU ZIKV-BR 4 weeks after DNA-M.ENV and ZPIV vaccination and 8 weeks after RhAd52-M.ENV vaccination. (B-F) Log MN50 titers against DENV serotypes 1–4, JEV, YFV and ZIKV induced by each DENV-PIV vaccine. (B) DENV-1, (C) DENV-2, (D) DENV-3, (E) DENV-4, and (F) TDENV-LAV. (G-I) Log anti-ZIKV MN50 titers in mice previously exposed or not to DENV vaccines in (G) DNA-M.ENV, (H) ZPIV, and (I) RhAd52-M.ENV vaccinated mice. Data is representative of one experiment with 4–5 animals per group. Each dot represents an individual mouse.

We then investigated the effect of pre-immunization with one of the individual serotype DENV PIVs or the tetravalent TDENV-LAV vaccine on ZIKV vaccine efficacy in BALB/c mice [22]. Groups of mice (N = 5 per group) were pre-immunized with 1 μg purified (1) DENV-1 PIV, (2) DENV-2 PIV, (3) DENV-3 PIV, (4) DENV-4 PIV or with (5) 10^2^ PFU of TDENV-LAV at week 0. At week 4, mice were immunized with the following ZIKV vaccines: (a) 10^9^ viral particles of rhesus adenovirus 52-M-ENV (RhAd52-M.ENV), (b) 50 μg of DNA-M-ENV or (c) 1 μg GMP ZPIV. Mice that received the DNA-M-ENV and the ZPIV vaccines received an additional boost at week 8 (Fig 3A).

Mice vaccinated with the DENV-1 PIV vaccine showed robust NAb responses against DENV-1 and DENV-3 (Fig 3B). DENV-2 PIV vaccine induced robust NAbs responses against DENV-1, DENV-2, and DENV-3 (Fig 3C). DENV-3 PIV vaccine-induced NAbs responses against DENV-1 and DENV-3, DENV-4 PIV induced NAbs responses against DENV-1, DENV-2, and DENV-3, but only weakly against DENV-4 (Fig 3D–3E). TDENV-LAV induced cross-reactive NAbs responses primarily against DENV-1 and DENV-3 (Fig 3F). None of the DENV vaccines induced detectable cross-reactive NAbs against ZIKV, Japanese encephalitis virus (JEV), or YFV (Fig 3B–3F). The DNA, ZPIV, and RhAd52 based ZIKV vaccines induced Zika NAbs that were not enhanced or suppressed by pre-existing anti-DENV immunity (Fig 3G–3I).

**Fig 4:**
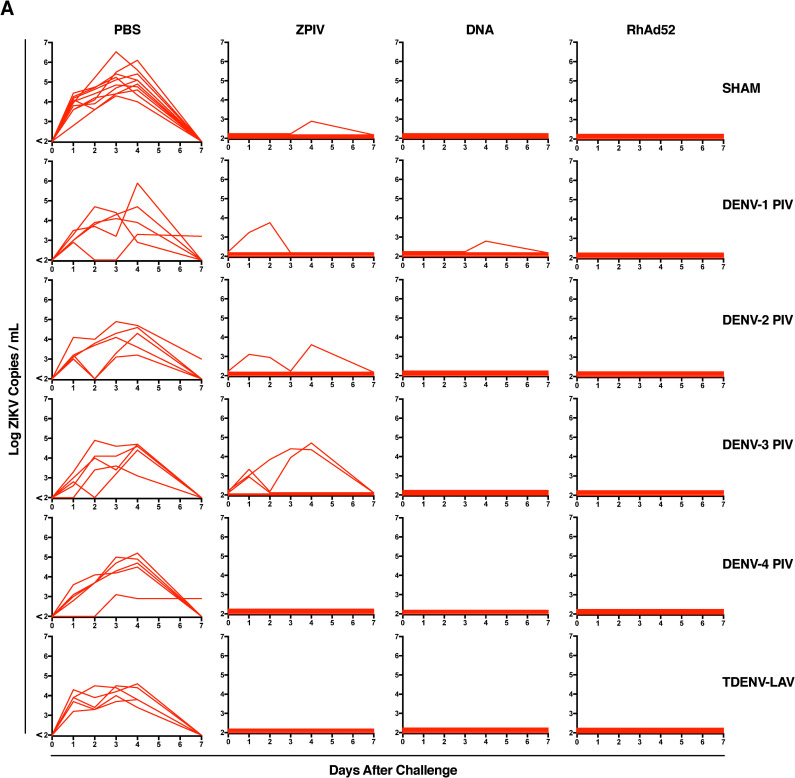
Previous DENV-Immunity induced by vaccination does not enhance ZIKV disease in mice. Mice were immunized intramuscularly (i.m.) on weeks 0 with 1 μg of each DENV serotype of the purified inactivated vaccine (DENV-PIV) or 10^2^ PFU of the live attenuated tetravalent DENV vaccine (TDENV-LAV) and vaccinated on weeks 4 and 8 with 50 μg of the DNA-M.ENV vaccine and 1 μg of the purified inactivated ZIKV (ZPIV). Mice received a single immunization with 10^9^ vp of the RhAd52-M.ENV vaccine. Mice were challenged i.v. with 10^2^ PFU ZIKV-BR 4 weeks after DNA-M.ENV and ZPIV vaccination and 8 weeks after RhAd52-M.ENV vaccination. (A) ZIKV mRNA viral loads in serum. Data is representative of one experiment with 5–10 animals per group. Each line represents an individual mouse.

Next, all mice were challenged with 10^2^ PFU of ZIKV-BR by the intravenous (i.v.) route at week 12, reflecting 4 weeks after the ZPIV and DNA-M.ENV boost or 8 weeks after the single-shot RhAd52-M.ENV vaccination. Sham vaccinated mice had high levels of ZIKV RNA in serum following challenge (Fig 4). Overall, ZPIV and DNA vaccination protected most animals from ZIKV infection, with several breakthrough infections observed in ZPIV vaccinated mice and one breakthrough in DNA vaccinated mice. Mice that received the RhAd52 vaccine were completely protected against ZIKV challenge. These data suggest that DENV immunity acquired through immunization had little effect on ZIKV disease enhancement and did not abrogate ZIKV vaccine protection in mice using multiple vaccine platforms.

### Passive transfer of DENV-specific IgG into *STAT2* ^-/-^ mice does not enhance ZIKV infection

**Fig 5:**
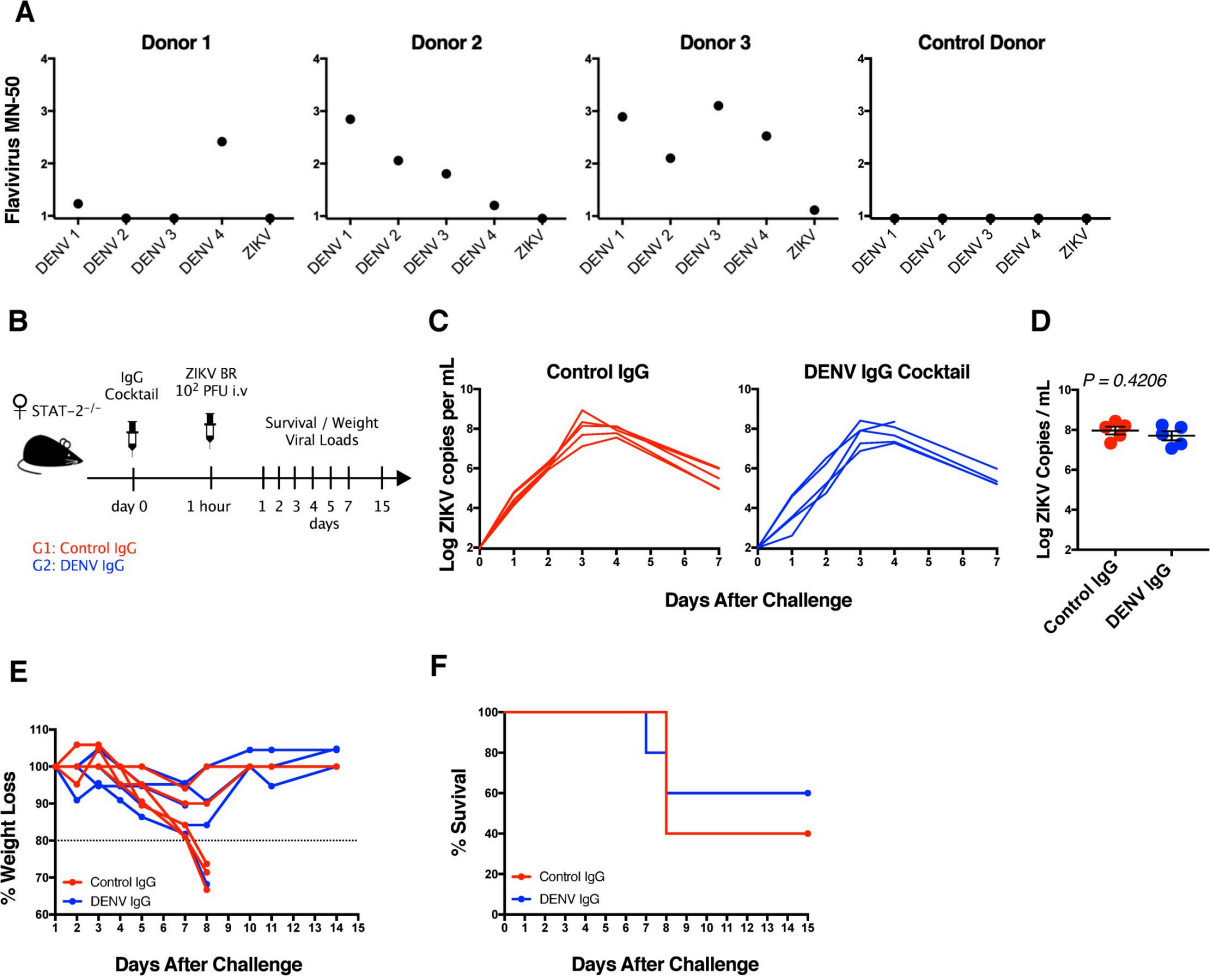
Passive transfer of anti-DENV IgG in STAT-2^-/-^ mice. Anti-DENV IgG (200 μg) isolated from 3 donors were pooled and passively transferred into *STAT2*^-/-^ mice. (A) MN50 for previous flavivirus infection in 3 DENV infected donors and 1 control donor. One-hour post IgG transfer, mice were challenged i.v. with 10^2^ PFU ZIKV-BR. (B) Schematic design of the experiment. (C) ZIKV mRNA viral load in control-IgG and DENV-IgG recipients. (D) Peak viral load comparison in control-IgG vs DENV-IgG recipients. (E) Percentage weight loss in mice post ZIKV challenge. (F) Percentage of mice survival post ZIKV challenge. Data is representative of one experiment with n = 5 animals per group. Each line represents an individual mouse. *P* values were calculated using a *Mann-Whitney U test*. Mean ± SEM is shown.

A previous study reported that passive transfer of plasma from DENV immune patients into *STAT2* ^-/-^ mice enhanced ZIKV infection, and this led us to ask whether plasma anti-DENV IgG was responsible for disease enhancement [17]. To address this question, IgG was purified from convalescent plasma from three humans who were previously infected with DENV and then passively transferred into *STAT2* ^-/-^ mice, in the C57BL/6 background, prior to ZIKV challenge. Donor 1 had NAbs against DENV-4; donor 2 had NAbs against DENV-1, DENV-2, and DENV-3; and donor 3 had NAbs against all four DENV serotypes (Fig 5A). None of the donors exhibited NAbs against ZIKV. In addition, we purified IgG from a control donor who had no detectable NAbs against either DENV or ZIKV (Fig 5A). We pooled IgG from all three donors and passively transferred 200 μg of the DENV-specific IgG or control IgG into two groups of *STAT2* ^-/-^ mice (n = 5) by the i.v. route. At 1 hour following passive transfer, mice were challenged by the i.v. route with 10^2^ PFU ZIKV-BR (Fig 5B). We observed comparable viremia in mice that received DENV-specific or control IgG (Fig 5C), and no differences were observed at peak viremia on day 3 (Fig 5D). Mice from both groups exhibited rapid weight loss (Fig 5E), and 2 of 5 (40%) mice that received DENV-specific IgG and 3 of 5 (60%) mice that received control IgG succumbed to infection by day 8 post ZIKV infection (Fig 5E and 5F).

**Fig 6:**
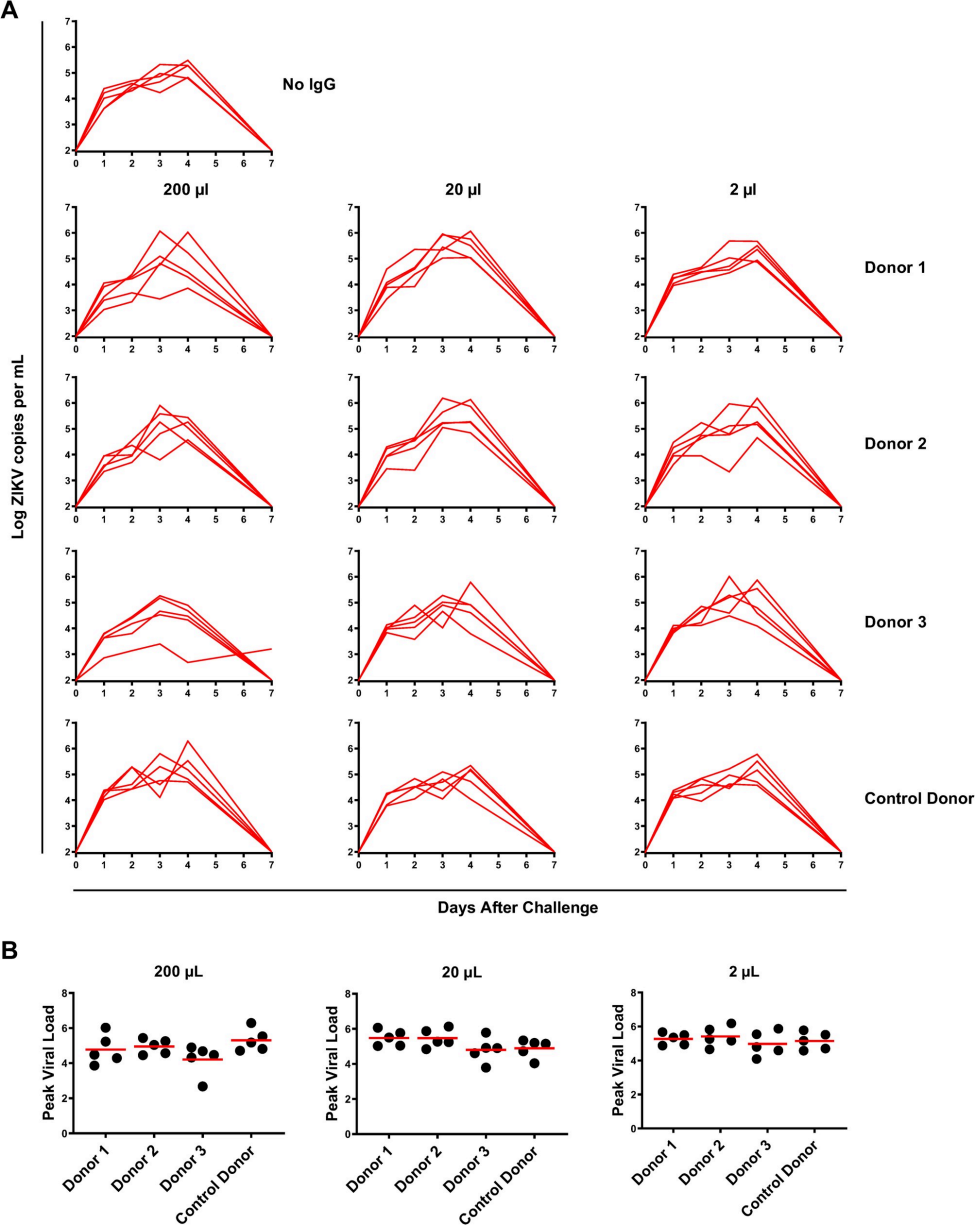
Passive transfer of anti-DENV IgG in BALB/c mice. Anti-DENV IgG isolated from 3 donors were passively transferred into BALB/c mice in 3 different doses (200 μl, 20 μl, and 2μl) equivalent of (200 μg, 20 μg and 2 μg) dose. One-hour post IgG transfer, mice were challenged i.v. with 10^2^ PFU ZIKV-BR. (A) ZIKV mRNA viral load in serum. (B) Peak viral load comparison in all recipients. Data is representative of one experiment with n = 5 animals per group. All injections were performed in the final volume of 200 μl diluted in saline. Each line or dot represents an individual mouse.

Finally, we assessed if the overall concentration of DENV-specific IgG may influence ZIKV infection. Four groups of BALB/c mice (n = 5) were infused with a low, intermediate, and high dose (2 μl, 20 μl, and 200 μl, respectively) of 10 mg/ml pooled donor IgG or control IgG and challenged i.v. with 10^2^ PFU of ZIKV-BR. No difference in ZIKV viremia was observed by any of these doses of DENV-specific IgG when compared to each other or the control group (Fig 6). These data suggest that the previously reported negative impact of DENV-specific plasma on ZIKV challenge may not be due to pre-existing DENV-specific IgG against certain DENV serotypes, and a more comprehensive study is needed to address this outcome.

## Discussion

In this study, we assessed the impact of baseline DENV-specific immunity on subsequent infection with ZIKV and ZIKV vaccine efficacy in both rhesus macaques and mice. We found that pre-immunization with DENV vaccines induced robust DENV-specific NAbs but did not significantly impact viral replication following ZIKV challenge. In addition, we did not observe a reduction in immunogenicity or protective efficacy of candidate ZIKV vaccines, including ZPIV in macaques and DNA, ZPIV, and RhAd52 vaccines in mice. Moreover, passive transfer of purified DENV-specific IgG from three convalescent human donors did not enhance ZIKV infection in mice. These data demonstrate that vaccine-induced or naturally induced DENV immunity did not exacerbate ZIKV infection and did not compromise ZIKV vaccination in these models.

Immunological cross-reactivity has been hypothesized to lead to antibody-dependent enhancement (ADE) between heterotypic DENV strains and other flaviviruses *in vitro* as well as in susceptible small animal models [9–11,13–17]. Whether DENV-specific IgG can worsen ZIKV infection remains unclear. We did observe a modest but not statistically significant elevation in ZIKV viral loads in both plasma and tissues in a small subset of animals previously immunized the live-attenuated tetravalent DENV vaccine (TDENV-LAV). In mice, this effect was less apparent than in the macaque study, and breakthrough ZIKV viremia was only observed in a small subset of mice not previously vaccinated with TDENV-LAV compared to what was observed in the macaques. Breakthrough viremia was observed in one mouse in both the DENV-1 and DENV-2 PIV pre-immunization groups and two mice for the DENV-3 PIV immunization groups (Fig 4). In combination with evidence for strong cross-reactive anti-ZIKV endpoint titers following DENV-1, DENV-2, and DENV-3 PIV pre-immunization in the same mouse strain, one might speculate a marginal role in ADE in modulating vaccine protection following ZIKV vaccination and subsequent challenge in these mice. However, ADE is unlikely to influence the elevated ZIKV viremia observed in both macaques and BALB/c mice. No breakthrough viremia was observed in mice immunized with RhAd52 ZIKV vaccines in any DENV vaccine pre-immunization group and pre-immunization with DENV-4 PIV even though high cross-reactive anti-ZIKV IgG was detected in mice preimmunized with DENV-4 PIV (Figs 4 and S2). In addition, ZIKV viral loads did not increase in ZIKV challenged *STAT2* ^-/-^ mice passively transferred with human anti-DENV IgG prior to challenge. Furthermore, the GMP ZPIV preparation used in this study was less immunogenic than previous preparations of research-grade ZPIV [21,22]. These data suggest that suboptimal vaccine protection is a more likely explanation for incidences of breakthrough viremia. It is important to note that other studies have detected elevated ZIKV viral loads after challenge in both plasma and tissues, following passive transfer of anti-DENV immune sera from humans or previously DENV immunized BALB/c mice into both *STAT2*^*-/-*^ and CD11c-*Ifnar1*^-/-^ mouse models [23]. These data attest that the specific mouse strain used, due to differences in pathogenesis, viral tropism, and susceptibility, may affect the magnitude of ZIKV disease enhancement observed. Thus, further studies performed with different ZPIV preparations in multiple animal models are required to determine whether the increased ZIKV viremia observed in animals previously vaccinated with DENV vaccines is a reproducible outcome.

Although the discussion regarding ADE has concentrated primarily on the presence of cross-reactive antibodies, ADE is better understood as the result of an intricate combination of immune factors, including not only antibody affinity but also the precise masking of critical surface epitopes required for viral entry, a defining feature for whether an antibody at sufficient concentrations is neutralizing or sub-neutralizing [24]. In addition, physical parameters, such as binding angle and stoichiometry, also play a role in determining whether an antibody sufficiently binds to viral particles at neutralizing or sub-neutralizing concentrations. Furthermore, the degree to which different Fcγ receptors are engaged, i.e., FcγRI and FcγRII, also contribute to ADE severity [25]. As important as binding affinity is to cross-reactivity between flaviviral lineages, additional features should also be investigated in future DENV and ZIKV co-vaccination studies involving ADE.

ZIKV infection has garnered global interest due to its ability to cross the placenta and cause clinically severe birth defects, such as microcephaly in developing fetuses. Multiple reports have claimed that pre-existing anti-DENV IgG may not only exacerbate vertical ZIKV transmission between mother and fetus but also enhance ZIKV infection in fetal tissue via Fcγ-receptor-mediated ADE [26–29]. Although adenoviral vector delivery of Ad26 and RhAd52 ZIKV M-Env vaccines have conferred potent maternal-fetal protection in *Ifnabr*^-/-^ mouse models, whether prior DENV immunization with multiple vaccine regimens significantly enhances subsequent ZIKV fetal transmission and infection is still unknown [30]. We did not evaluate ZIKV transmission between the maternal-fetal interface or measure ZIKV titers in fetal tissues in pregnant female mice in our study. Therefore, we were unable to establish whether vaccine-induced pre-existing DENV immunity with or without subsequent ZIKV vaccine boost elicited any deleterious side effects on fetal development. Additional studies would be required to address this question.

We show that purified DENV-specific IgG derived from three DENV-experienced human donors did not enhance ZIKV infection in susceptible *STAT2* ^-/-^ mice. These data contrast with a previous report showing that DENV-specific plasma led to an increase in ZIKV viral load and pathogenesis in *STAT2* ^-/-^ mice following ZIKV challenge [17]. However, in this prior report, studies with purified IgG were not performed, and thus it is not clear if the observed effects are related to antibodies or other plasma components. In addition, the methodologies between our study and this study differed in some respects; for example, inoculations were performed intradermally as opposed to intravenously in our study, a different ZIKV strain was used, and an overall higher titer was administrated (5 × 10^3^ PFUs of ZIKV strain PRVABC59 compared to 100 PFUs of ZIKV-BR in our study). These factors would have also contributed to any differences between the studies. Another prior study in rhesus macaques, in agreement with our findings, demonstrated that baseline DENV-specific immunity did not enhance ZIKV infection following challenge [31]. Our study confirms and extends this prior study by showing that the immunogenicity and protective efficacy of candidate ZIKV vaccines do not appear to be compromised by baseline DENV-specific immunity in rhesus macaques and mice.

In summary, we show that pre-immunization with DENV vaccines to induce DENV-specific immunity did not exacerbate subsequent ZIKV infection or compromise the protective efficacy of candidate ZIKV vaccines in rhesus macaques and mice. These findings can help inform vaccination strategies against multiple pathogenic flaviviruses, and further studies of ZIKV vaccines in DENV-experienced humans are warranted.

## Material and methods

### Ethics statement

All animal studies were approved by the Bioqual Institutional Animal Care and Use Committee (IACUC). All experiments conformed to regulatory standards outlined by the American Veterinary Medical Association (AVMA) and the American Association of Laboratory Animal Medicine (AALAM).

### Animals, vaccines, and challenges

BALB/c and *STAT2* ^-/-^ female mice at 6–8 weeks of age were purchased from Jackson Laboratories (Bar Harbor, ME, USA). Mice were vaccinated with 50 μg DNA vaccine in saline without adjuvant, 10^9^ vp of RhAd52 vaccine in saline without adjuvant, 1 μg of DENV/ZIKV purified inactivated vaccines (PIV) (derived from DENV-1 strain West Pac 74, DENV-2 strain S16803, DENV-3 strain CH53489, DENV-4 TVP-360, and ZIKV isolate PRVABC59) with 100 μg alum (Alhydrogel; Brenntag Biosector, Denmark), or 10^2^ PFU of the fully formulated tetravalent live attenuated (TDENV-LAV) a gift from the Walter Reed Army Institute of Research (WRAIR, lot #1856) by the i.m. routes in a 100 μl volume [32]. PIV vaccines and TDENV-LAV were generated and inactivated or attenuated as described previously [22,32,33]. In brief, DENV and ZIKV PIV were passaged through Vero cells and inactivated via 0.05% formalin treatment for seven days, and TDENV-LAV was attenuated by serial passage through primary dog kidney cell culture [22,32,33]. Mice were then challenged by the i.v. route with 10^2^ plaque-forming units (PFU) ZIKV-BR strain [22,34]. Animals were randomly allocated to groups. Immunologic and virologic assays were performed blinded. All animal studies were approved by the BIDMC Institutional Animal Care and Use Committee (IACUC).

32 outbred, Indian-origin male and female rhesus monkeys (*Macaca mulatta*) were housed at Bioqual, Rockville, MD. Monkeys were immunized by the s.c. route with 5 μg purified ZIKV PIV (ZPIV) with alum (Alhydrogel; Brenntag Biosector) or 10^3^ PFU of the live attenuated DENV vaccine TDENV-LAV by the s.c. route. Monkeys were then challenged by the s.c. route with 10^3^ plaque-forming units (PFU) ZIKV-BR [22].

### RT-PCR

RT-PCR assays were utilized to monitor viral loads in plasma, cerebrospinal fluid, cervicovaginal swab, colorectal swab, urine and lymph node. RNA was extracted with a QIAcube HT (Qiagen, Germany). Serum samples were extracted using the cador Pathogen 96 QIAcube HT Kit, and tissue samples were lysed in Qiazol, using the Tissuelyser II (Qiagen, Germany), chloroform treated and extracted with the RNeasy 96 QIAcube HT Kit. The wild-type ZIKV BeH815744 Cap gene was utilized as a standard. RNA standards were generated using the AmpliCap-Max T7 High Yield Message Maker Kit (Cell Script) and purified with RNA clean and concentrator kit (Zymo Research, CA, USA). RNA quality and concentration were assessed by the BIDMC Molecular Core Facility. Log dilutions of the RNA standard were reverse transcribed and included with each RT-PCR assay. Viral loads were calculated as RNA copies per milliliter or VP per microgram of total RNA as measured on the NanoDrop (Thermo Scientific, Waltham, MA, USA). Assay sensitivity was >100 copies/ml and > 1 copy/μg total RNA.

### MN50 Microneutralization assay

A high-throughput ZIKV microneutralization (MN) assay was used for measuring ZIKV-specific neutralizing antibodies, as previous described [21,22]. Briefly, serum samples were serially diluted threefold in 96-well microplates, and 100 μl of ZIKV-PR (PRVABC59) containing 100 PFU was added to 100 μl of each serum dilution and incubated at 35°C for 2 hours. Supernatants were then transferred to microtiter plates containing confluent Vero cell monolayers (World Health Organization, NICSC-011038011038). After incubation for 4 days, cells were fixed with absolute ethanol/methanol for 1 hour at −20°C and washed three times with PBS. The pan-flavivirus monoclonal antibody 6B6-C1 conjugated to HRP (6B6C-1 was a gift from J. T. Roehrig, U.S. Centers for Disease Control and Prevention) was then added to each well, incubated at 35°C for 2 hours, and washed with PBS. Plates were washed, developed with TMB for 50 min at room temperature, and stopped with 1:25 phosphoric acid, and absorbance was read at 450 nm. For a valid assay, the average absorbance at 450 nm of three noninfected control wells had to be ≤ 0.5, and virus-only control wells had to be ≥ 0.9. Normalized absorbance values were calculated, and the MN50 titer was determined by a log midpoint linear regression model. The MN50 titer was calculated as the reciprocal of the serum dilution that neutralized ≥ 50% of ZIKV, and seropositivity was defined as a titer ≥ 10, with the maximum measurable titer of 7290. Log10 MN50 titers are reported.

### Passive antibody transfer

Polyclonal immunoglobulin G (IgG) was individually purified with protein G purification kits (Thermo Fisher Scientific, MA) from 3 DENV positive donor human plasma and one control. De-identified human plasma without any protected health information were obtained from Dr. Michael Busch at the University of California, San Francisco. Total IgG was buffer-exchanged into 1× PBS according to methods. Purified IgG was infused intravenously into groups of naïve recipient BALB/c or *STAT2* ^-/-^ mice before ZIKV-BR challenge (10^2^ plaque-forming units (PFU)) at 1 hour after infusion. Groups of 5 mice received de-escalating doses (200 μl, 20 μl, or 2 μl) of a 10 mg/ml solution of purified IgG.

### Statistical analyses

Analysis of virologic and immunologic data was performed using GraphPad Prism v6.03 (GraphPad Software). Comparisons of groups were performed using t-tests and two-way analysis of variance with Tukey’s test for multiple comparisons.

## Supporting information

S1 Fig Peak ZIKV plasma viral loads in BALB/c mice inoculated via the intravenous (i.v.) or subcutaneous (s.c.) with PFU of ZIKV over the course of one week. (A-B) Plasma viral loads in mice inoculated both i.v. (A) and s.c. (B) after a one-week infection course. (C) Peak viral load between mice in the i.v. and s.c. groups. Statistical significance was calculated using a Mann-Whitney U test.
(TIF)

S2 Fig Anti-ZIKV endpoint titers following pre-immunization with different DENV vaccines. BALB/c mice were prime immunized with the corresponding serotype or tetravalent DENV vaccine and boosted four weeks later with each respective ZIKV vaccine (RhAd52, ZPIV, or DNA). Anti-ZIKV endpoint titers were measured four weeks following ZIKV vaccination, eight weeks following DENV vaccine prime. The sham group received no ZIKV vaccine aund naïve mice were given neither the DENV or ZIKV vaccine.
(TIF)
