# Supplementary figures and images for: Impact of prior Dengue immunity on Zika vaccine protection in rhesus macaques and mice
Source: PLoS Pathog. 2021 Jun 25;17(6):e1009673. doi: 10.1371/journal.ppat.1009673 (PMC8266125; doi:10.1371/journal.ppat.1009673)

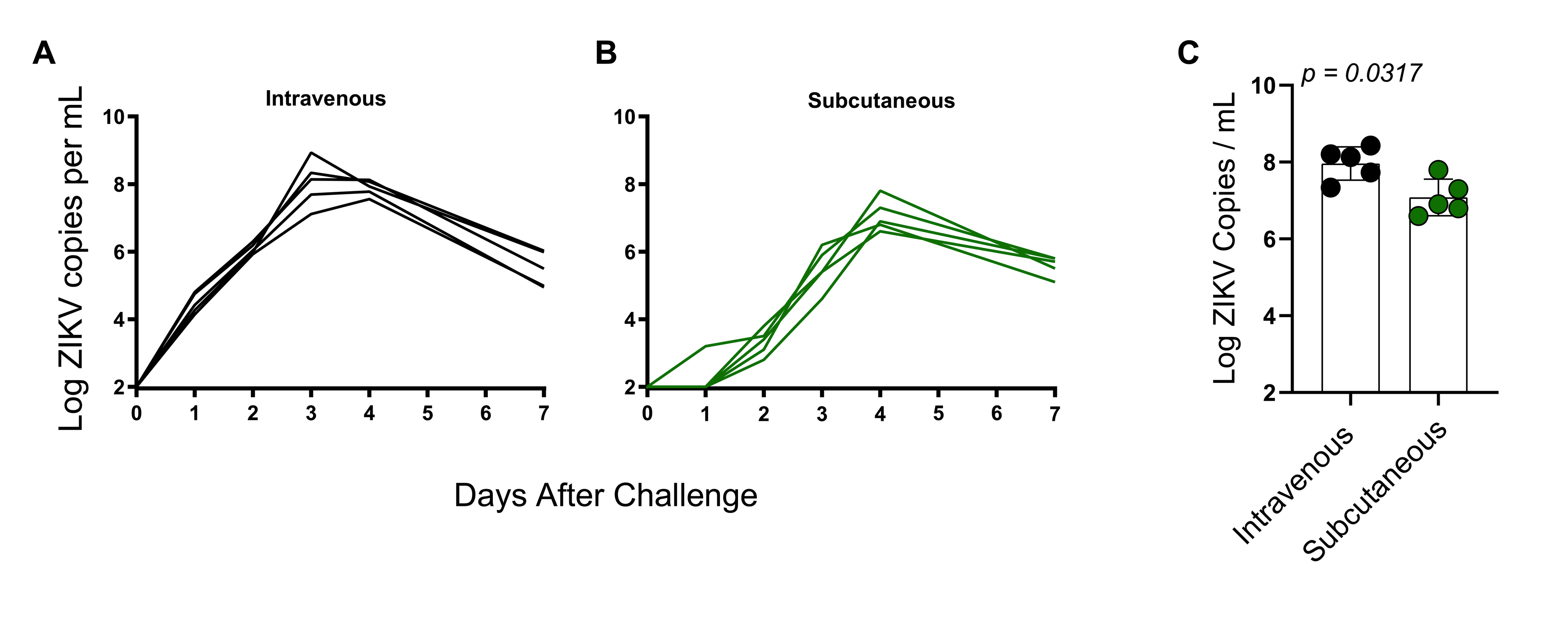

Supplement: S1 Fig — (A-B) Plasma viral loads in mice inoculated both i.v. (A) and s.c. (B) after a one-week infection course. (C) Peak viral load between mice in the i.v. and s.c. groups. Statistical significance was calculated using a Mann-Whitney U test. (TIF) [file ppat.1009673.s001.tif]

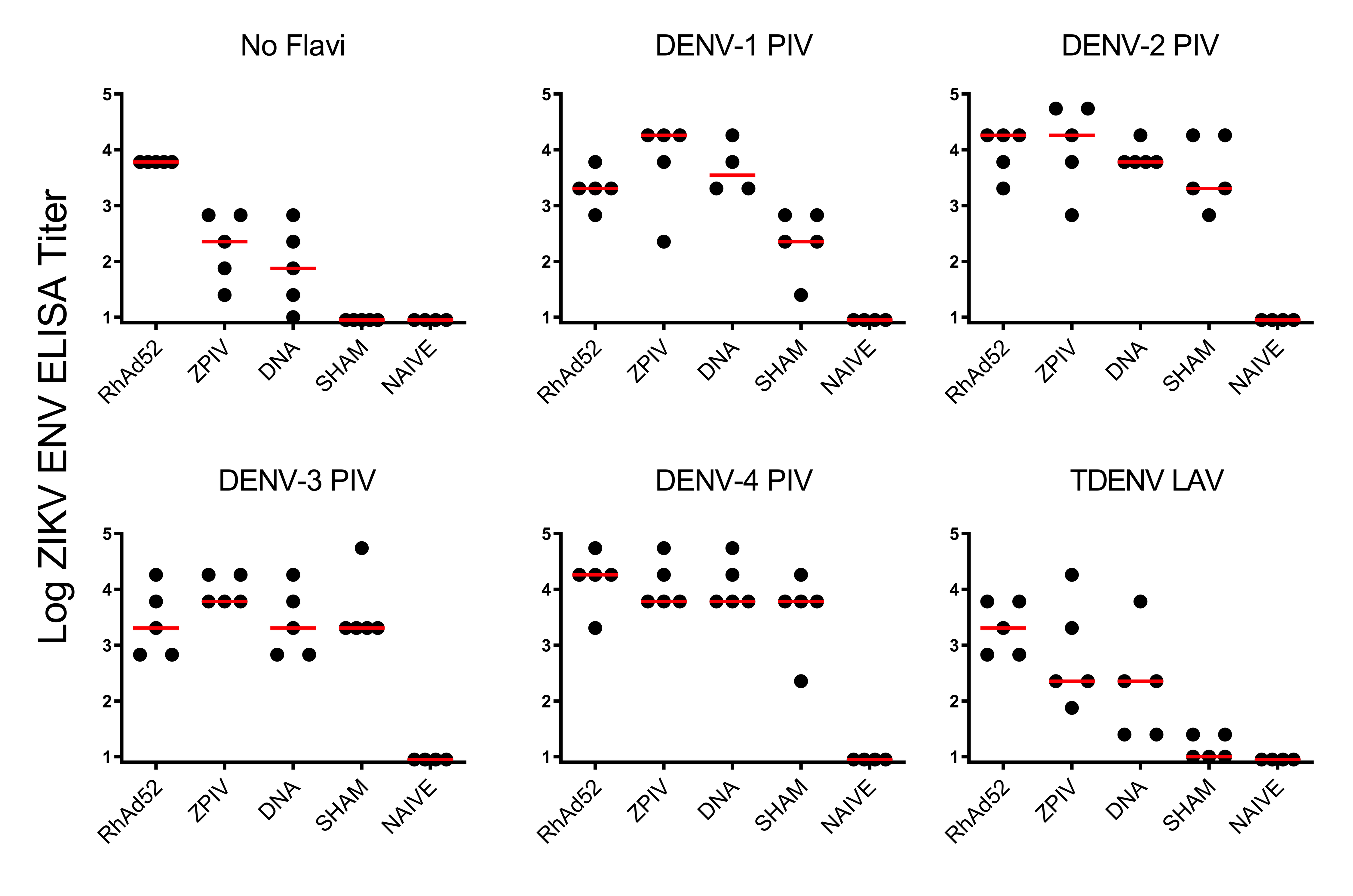

Supplement: S2 Fig — BALB/c mice were prime immunized with the corresponding serotype or tetravalent DENV vaccine and boosted four weeks later with each respective ZIKV vaccine (RhAd52, ZPIV, or DNA). Anti-ZIKV endpoint titers were measured four weeks following ZIKV vaccination, eight weeks following DENV vaccine prime. The sham group received no ZIKV vaccine aund naïve mice were given neither the DENV or ZIKV vaccine. (TIF) [file ppat.1009673.s002.tif]
